# Evolution of Variable Number Tandem Repeats and Its Relationship with Genomic Diversity in *Salmonella* Typhimurium

**DOI:** 10.3389/fmicb.2016.02002

**Published:** 2016-12-26

**Authors:** Songzhe Fu, Sophie Octavia, Qinning Wang, Mark M. Tanaka, Chin Yen Tay, Vitali Sintchenko, Ruiting Lan

**Affiliations:** ^1^School of Biotechnology and Biomolecular Sciences, University of New South Wales (UNSW)Sydney, NSW, Australia; ^2^Centre for Infectious Diseases and Microbiology–Public Health, Institute of Clinical Pathology and Medical Research, Westmead HospitalSydney, NSW, Australia; ^3^Pathology and Laboratory Medicine, University of Western AustraliaPerth, WA, Australia; ^4^Marie Bashir Institute for Infectious Diseases and Biosecurity, University of SydneySydney, NSW, Australia

**Keywords:** *S*. Typhimurium, SNP, VNTR, directional mutability, epidemiological typing

## Abstract

*Salmonella enterica* serovar Typhimurium is the most common *Salmonella* serovar causing human infections in Australia and many other countries. A total of 12,112 *S*. Typhimurium isolates from New South Wales were analyzed by multi-locus variable number of tandem repeat (VNTR) analysis (MLVA) using five VNTRs from 2007 to 2014. We found that mid ranges of repeat units of 8–14 in VNTR locus STTR5, 6–13 in STTR6, and 9–12 in STTR10 were always predominant in the population (>50%). *In vitro* passaging experiments using MLVA type carrying extreme length alleles found that the majority of long length alleles mutated to short ones and short length alleles mutated to longer ones. Both data suggest directional mutability of VNTRs toward mid-range repeats. Sequencing of 28 isolates from a newly emerged MLVA type and its five single locus variants revealed that single nucleotide variation between isolates with up to two MLVA differences ranged from 0 to 12 single nucleotide polymorphisms (SNPs). However, there was no relationship between SNP and VNTR differences. A population genetic model of the joint distribution of VNTRs and SNPs variations was used to estimate the mutation rates of the two markers, yielding a ratio of 1 VNTR change to 6.9 SNP changes. When only one VNTR repeat difference was considered, the majority of pairwise SNP difference between isolates were 4 SNPs or fewer. Based on this observation and our previous findings of SNP differences of outbreak isolates, we suggest that investigation of *S*. Typhimurium community outbreaks should include cases of 1 repeat difference to increase sensitivity. This study offers new insights into the short-term VNTR evolution of *S*. Typhimurium and its application for epidemiological typing.

## Introduction

All bacterial genomes contain repetitive DNA in multiple loci called variable-number tandem-repeat (VNTR) (van Belkum, 2007). Due to their high variability and ease of detection, VNTRs are useful markers for epidemiological studies. Multilocus VNTR analysis (MLVA), has been established for many bacterial pathogens with epidemic potential, including *Yersinia pestis* (Klevytska et al., 2001), *Salmonella enterica* serovar Typhimurium (Lindstedt et al., 2004a), *Escherichia coli* O157 (Lindstedt et al., 2004b) and has been applied to trace chains of ongoing disease transmission (Lindstedt et al., 2013) and for outbreak investigations (Nadon et al., 2013).

In Australia, *S*. Typhimurium accounts for the majority of human *Salmonella* infections. The 5-locus MLVA scheme with the following locus order: STTR9-STTR5-STTR6-STTR10-STTR3, has been adopted as a standardized method for routine surveillance of *S*. Typhimurium in the state of New South Wales (NSW), Australia (Sintchenko et al., 2012). However, since a small number of MLVA types dominate, which we refer to as endemic MLVA types, the discriminatory power of MLVA is significantly reduced for outbreak investigations when they are caused by endemic MLVA types as the same MLVA type can potentially be found in epidemiologically unlinked cases (Sintchenko et al., 2012). Spatial and temporal clustering can be used to exclude epidemiologically unlinked cases of the same MLVA type. In our current practice, recovery of five or more geographically clustered isolates of the same MLVA type within a 4-week window period has been used as a trigger for public health investigation (Sintchenko et al., 2012).

VNTRs can mutate rapidly; parallel or reverse changes can occur at the same locus leading to the same MLVA types with no common recent ancestry (Wuyts et al., 2013; Dimovski et al., 2014; Ahlstrom et al., 2015). MLVA types with one repeat difference are considered as the most closely related and relationships of MLVA types with multiple allele differences often do not reflect true phylogenetic relationships of the isolates (Octavia and Lan, 2009; Lam et al., 2012). MLVA is not a suitable typing tool for long-term epidemiology.

The stepwise mutation model (SMM) (Ohta and Kimura, 1973) was the first and simplest model of stepwise change with mutations adding or deleting a single unit, and this model can be used to describe VNTR evolution. The model assumes a fixed rate of random mutations and insertion and deletion have equal probability of occurrence. Vogler et al. (2006) observed initially in *E. coli* that the frequency of mutations depends on the number of repeats involved and only for small repeat number mutations insertions and deletions occur at equal frequencies. A geometric model was developed to account for the differences in the relative frequency of multiple-repeat mutations. This model was generalized to other bacterial species (Vogler et al., 2007). Similarly, Aandahl et al. (2012) found that the linear model of VNTR mutation rate with respect to repeat numbers fits better than a constant mutation rate model in *Mycobacterium tuberculosis*. However, these models did not consider the direction of change as a function of repeat number.

There is a need for better understanding of the evolutionary dynamics of repeat changes in VNTRs and the relationship between VNTR differences and genomic differences in order to infer true genetic relationships between similar MLVA types for epidemiological typing. In this study we examined the patterns of mutations of VNTR repeats from isolates recovered from human and environmental sources and also from *in vitro* laboratory passaging experiments and demonstrated that VNTRs mutated directionally toward repeat units of median size. We also sequenced 28 isolates from a newly emerged MLVA type and its closely related MLVA types to examine the relationship between VNTR and SNP variation.

## Materials and methods

### MLVA data and selection of strains for genome sequencing

The MLVA-5 data for 12,112 *S*. Typhimurium isolates, which were obtained during routine clinical diagnosis and submitted to the NSW Enteric Reference Laboratory, Pathology West, Sydney for serotyping and MLVA typing in 2007–2014, were analyzed (Sintchenko et al., 2012).

To observe natural VNTR evolution, strains from a novel MLVA type 2-15-8-10-212 (European CDC convention) and its related MLVA types, which emerged from June 2012, were randomly sampled every 2–3 months. Each sampling consisted of strains with MLVA type 2-15-8-10-212 and the MLVA types with one repeat difference which were isolated within 1 month (if available) to represent the diversity of these closely related MLVA types from June 2012 to April 2014. To reduce the impact of geographical diversity, isolates from patients residing only in the eastern suburbs of Sydney were sampled. In total, 28 isolates from MLVA type 2-15-8-10-212 and its related MLVA types were selected for genome sequencing (Table 1, Table S1).

**Table 1:** **General features of ***S***. Typhimurium sequenced in this study**.

**Strain No**.	**Total No. of reads**	**N50**	**No. of Contigs**	**Total length (bp)**	**Mapping coverage**	**Isolation date**	**MLVA**
L2162	1,816,766	132,501	290	4,877,111	80.57	4/06/2012	2-15-8-10-212
L2197	2,530,368	113,011	275	4,887,162	110.71	26/07/2012	2-15-8-10-212
L2198	1,210,590	113,543	339	4,779,955	52.05	3/08/2012	2-15-8-10-212
L2199	1,683,656	256,437	296	4,881,238	63.41	19/12/2012	2-15-8-10-212
L2163	2,814,362	73,848	787	4,881,238	115.91	24/06/2012	2-14-8-10-212
L2164	2,240,932	40,859	791	4,921,427	90.75	3/08/2012	2-14-8-10-212
L2166	1,419,522	70,869	319	4,937,000	59.25	7/09/2012	2-15-8-10-212
L2167	1,896,018	114,482	279	4,924,157	81.73	11/11/2012	2-15-8-10-212
L2168	2,045,124	242,869	289	4,966,818	82.55	22/11/2012	2-14-8-10-212
L2169	1,715,494	225,223	304	4,980,861	68.58	4/02/2013	2-15-9-10-212
L2170	1,332,010	198,321	315	4,883,562	54.85	16/02/2013	2-15-8-10-212
L2171	1,036,414	247,240	267	4,886,167	44.43	25/02/2013	2-15-8-10-212
L2172	1,069,474	270,409	313	4,882,885	43.69	15/04/2013	2-15-9-10-212
L2173	2,687,050	149,266	269	4,878,027	120.43	29/04/2013	2-15-8-10-212
L2174	946,682	170,789	304	4,879,468	37.85	19/05/2013	2-15-8-11-212
L2175	1,488,622	242,867	282	4,885,894	77.94	30/08/2013	2-15-9-10-212
L2176	2,098,872	129,258	302	4,864,710	54.11	25/11/2013	2-15-8-10-212
L2177	955,134	213,895	240	4,883,749	45.36	25/11/2013	2-14-8-10-212
L2178	1,344,862	250,740	289	4,885,337	55.94	22/12/2013	2-15-9-10-212
L2179	2,271,256	222,083	261	4,884,431	92.54	11/01/2014	2-16-8-10-212
L2180	1,626,426	243,029	272	4,887,230	70.45	11/01/2014	2-15-7-10-212
L2181	1,859,206	113,847	321	4,888,447	81.27	27/01/2014	2-15-9-10-212
L2182	1,167,938	225,449	275	4,880,601	45.79	23/02/2014	2-15-8-10-212
L2183	1,505,900	246,520	267	4,886,352	61.57	26/02/2014	2-14-8-10-212
L2184	2,144,810	225,479	263	4,882,354	88.45	11/03/2014	2-15-8-11-212
L2185	1,128,550	174,736	278	4,880,851	42.44	1/04/2014	2-15-9-10-212
L2186	2,100,754	213,423	289	4,885,337	86.78	23/04/2014	2-15-7-10-212
L2187	2,050,606	243,038	274	4,882,961	89.32	29/04/2014	2-15-8-10-212
L1880	1,338,944	256,474	285	4,913,050	50.87	20/06/2011	2-12-10-10-212

### Genome sequencing, *de novo* assembly and identification of single nucleotide polymorphisms (SNPs)

The phenol/chloroform method was used to extract genomic DNA from each strain as described previously (Pang et al., 2013). Genomic DNA was sequenced using the Illumina Genome Analyzer (Illumina) with 300-bp paired-end sequencing. Contigs were *de novo* assembled using the Velvet version 1.0.8 and VelvetOptimiser (Zerbino and Birney, 2008) and were aligned to the *S*. Typhimurium LT2 genome (NC_003197, MLVA type 4-13-13-10-211) using progressiveMauve version 2.3.1 (Darling et al., 2010). The coverage of genomes was estimated by Qualimap v2.0 (Garcia-Alcalde et al., 2012). SNP calling was performed as described previously (Pang et al., 2013). A custom script was employed to extract SNPs according to the position on the reference genome. The raw genome sequence data from this study submitted to GenBank is under BioProject No. PRJNA348132.

### Phylogenetic analysis

Maximum likelihood phylogenetic trees were inferred using RAxML 7.2.8 (Stamatakis, 2006). Strain L1880 with MLVA type 2-12-10-10-212 was used as the background strain. Path-O-Gen v1.4 (Rambaut et al., 2016) and BEAST v1.8.2 (Drummond et al., 2012) were used for estimation of genome mutation. The raw estimates in units of substitutions per variable site per year based on a SNP alignment were scaled to genome-wide units of substitutions per site per year by multiplying the estimates by constant *k* = *n/N* where *n* is the number of SNP sites in the alignment and *N* is the total positions considered for SNP calling (4,487,272; Hawkey et al., 2013). Homoplasy of phylogenetic trees was measured by maximum parsimony analysis in PAUP4.0 (Swofford, 1993). Homoplasy of MLVA was defined as *1* − *(K* − *1)/M*, where *K* is the minimum number of MLVA mutation events and *M* is the actual number of VNTR changes along the SNP-tree (Chenal-Francisque et al., 2013).

### *In vitro* experimental evolution

Three independent replicates of strains L2162 and L2191 representing the two MLVA types, 2-15-8-10-212 and 2-26-6-19-112 respectively, were sub-cultured in Luria-Bertani (LB) liquid culture with two passages per day for a total of 50 passages as previously described (Wuyts et al., 2013). Each replicate was spread onto three LB agar plates and 33 colonies from each replicate were randomly selected. MLVA was performed on these colonies as described previously (Sintchenko et al., 2012).

### Relationship between SNP and VNTR mutations

To evaluate the relationship between SNP and VNTR mutations, pairwise comparisons between isolates were performed by observing SNP difference with a given VNTR locus or repeat difference as done by Eyre et al. (2013). Four pairwise comparisons were performed, including random pairwise comparisons between SNPs and different VNTR locus or repeat difference, pairwise comparisons based on the same time frame within a 1-month window and pairwise comparisons based on the lineages in the SNP tree. The range of SNP differences and the frequency of each SNP difference for all pairwise comparisons of zero, one and two VNTR repeat or locus changes using all VNTR loci were calculated.

### A model of the joint distribution of VNTR and SNP variation based on the coalescent

Let the mutation rate per genome per generation be denoted by λ_*G*_ and the mutation rate of the *i*th VNTR locus be denoted by μ_*i*_. To characterize the relationship between multilocus VNTR and SNP evolution, we also modeled the time separating two genomes via their most recent common ancestor using the standard coalescent model with an effective population size of *N* (for an introduction to coalescent theory, see Wakeley, 2008). In coalescent time units where 1 unit is *N* generations, assuming no homoplasy, the mutation rates generating new SNPs and MLVAs are

(1) $$\lambda =N\lambda_{G}$$

(2) $$\mu =N\sum \mu_{i}$$

Let *S* and *V* be the number of SNP and VNTR differences that evolve in these lineages respectively. We made a standard assumption that mutations along a branch occurred according to a homogeneous Poisson process so that over evolution time *t* the numbers of mutation of each type are distributed as Poisson (λ*t*) and Poisson (μ*t*). Assume that the mutations giving rise to SNPs and VNTRs are independent of each other along a given branch. We computed the joint distribution of the number of SNPs and MLVA differences in a data set as follows. The probability density of the total branch length *T* in a coalescent for *n* lineages is

(3) $$\begin{array}{c} f_{T}(t\text{​​}|\text{​​}n)=\underset{i=2}{\sum^{n}}{\left(-1\right)}^{i}\left(\begin{array}{c} n-1\\ i-1 \end{array}\right)\frac{i\;-\;1}{2}e^{-(i-1)t/2}. \end{array}$$

The joint distribution of the number of SNPs and MLVA differences is therefore given by

(4) $$\begin{array}{l} p(V=\upsilon ,S=s)=\int_{0}^{\infty }p(S=s|t)p(V=\upsilon |t)p(t)dt\\ \;=\int_{0}^{\infty }\frac{{\left(\lambda t\right)}^{s}{\left(\mu t\right)}^{\upsilon }e^{-(\lambda +\mu )t}}{s!\upsilon !}f_{T}(t|n)dt\\ \;=\frac{\lambda^{s}\mu^{\upsilon }}{s!\upsilon !}\sum_{i=2}^{n}{\left(-1\right)}^{i}\left(\begin{array}{c} n-1\\ i\;-\;1 \end{array}\right)\frac{i-1}{2}\\ \;\int_{0}^{\infty }t^{s+\upsilon }e^{-((i-1)/2+\mu +\lambda )t}dt \end{array}$$

where *p()* denotes probability mass. This can be further simplified by changing variables; define *x* = ((*i* − 1)/2 + μ + λ)*t* so that *dx*/*dt* = (*i* − 1)/2 + μ + λ. The integral in the above expression can then be rewritten as

(5) $$\begin{array}{c} \frac{\int_{0}^{\infty }x^{\upsilon +s}e^{-x}dx}{{\left(\frac{i-1}{2}+\mu +\lambda \right)}^{\upsilon \;+\;s\;+\;1}}=\frac{\Gamma (\upsilon +s+1)}{{\left(\frac{i-1}{2}+\mu +\lambda \right)}^{\upsilon \;+\;s\;+\;1}} \end{array}$$

Since υ, *s* are integer valued, the gamma function Γ(υ + *s* + 1) can be replaced with the factorial (υ + *s*)!, so that

(6) $$\begin{array}{l} p(V=\upsilon ,S=s)=\left(\begin{array}{c} \upsilon +s\\ \upsilon \end{array}\right)\lambda^{s}\mu^{\upsilon }\sum_{i\;=\;2}^{n}{\left(-1\right)}^{i}\left(\begin{array}{c} n-1\\ i-1 \end{array}\right)\\ \;\frac{\frac{i-1}{2}}{{\left(\frac{i-1}{2}+\mu +\lambda \right)}^{\upsilon \;+\;s\;+\;1}}. \end{array}$$

The maximum likelihood estimates of λ and μ can be obtained by numerically maximizing the above likelihood equation (Equation 6) with respect to those two parameters. The 95% confidence intervals (CI) for λ and μ were estimated using standard asymptotic theory for likelihood estimators under which standard errors used for the confidence interval are computed using the Fisher Information matrix (Casella and Berger, 2002). Any confidence limit below 0 was set to 0, and each CI includes a Dunn-Sidak correction for testing two hypotheses in a single dataset. The ratio of the two mutation rates (*r* = λ/μ) was estimated by re-parameterizing the model in terms of *r* and λ.

## Results

### Mutability and directionality of VNTR loci from analysis of clinical isolates over 8 years

**Figure 1:**
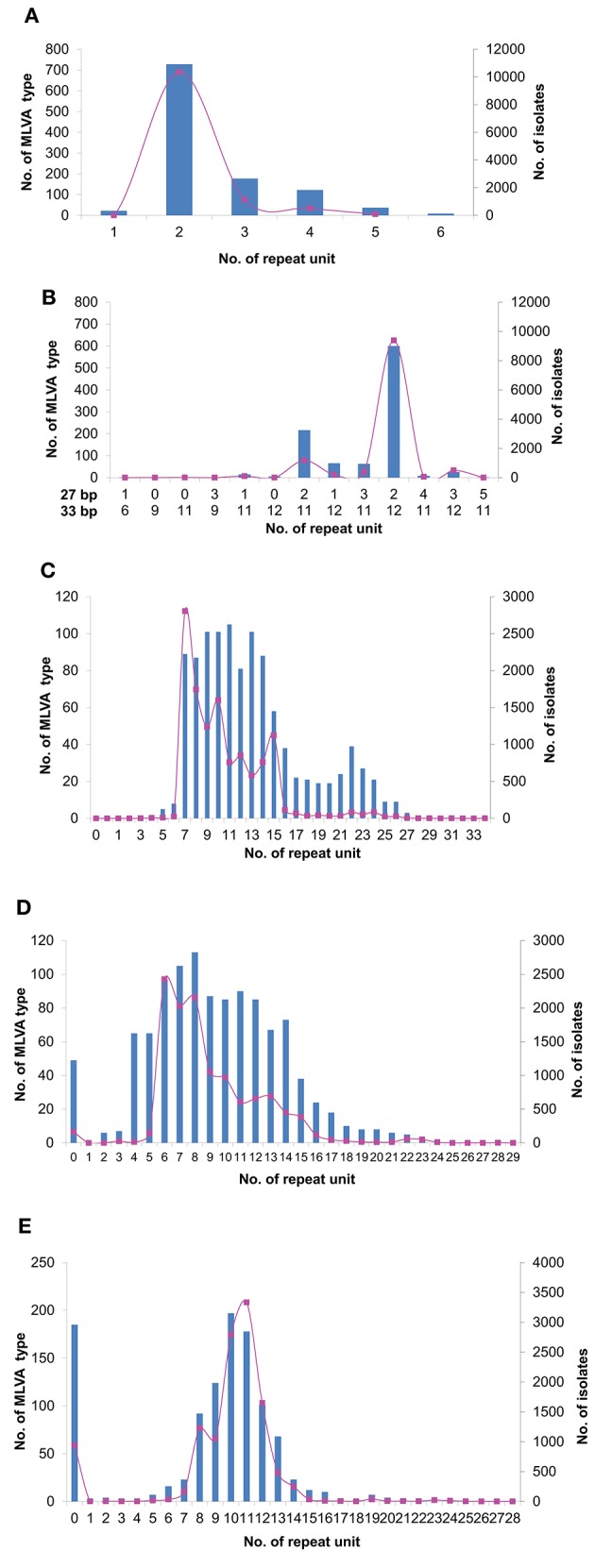
**Tendency of MLVA changes from 2007 to 2014**. Distribution of total number of isolates (pink lines) and MLVA types (blue bars) for VNTR loci STTR9 **(A)**, STTR3 **(B)**, STTR5 **(C)**, STTR6 **(D)**, and STTR10 **(E)**. For STTR3, the repeat unit of 27 and 33 bp were indicated below the X-axis, respectively.

We analyzed the allelic distribution of the MLVA data from 12,112 *S*. Typhimurium isolates from 2007 to 2014. For STTR9 and STTR3, allele 2 and allele 212 were predominant, accounting for 85.5% and 77.3% of the isolates respectively. For STTR9, the next most frequent alleles are 3 and 4 with 9.3% and 4.3% respectively. For STTR3, the next most frequent allele is 211 with 10.0%. There were only a few other alleles at these two loci with low frequencies (Figures 1A,B). STTR3 has a composite of two types of repeats with the unit length of 27 bp and 33 bp, making it more difficult to determine the repeat's composition. Additionally these two loci have been shown to be relatively stable from both *in vivo* and *in vitro* passaging experiments (Dimovski et al., 2014). Therefore, they were excluded from further analysis. For the remaining 3 VNTR loci, the distribution of the size of repeat units largely followed a normal distribution, with the median size having the highest frequency by both number of MLVA types and the total number of isolates (Figures 1C–E). We define mid-range repeats as those falling within the second and third quartiles (25–75%) of the repeat distribution. Any repeats outside the mid-ranges are considered to be extreme length repeats. The median number of repeats was 10 and the mid-range was 8–14 repeats for STTR5. Similarly, STTR6 had a median size of 8 and a mid-range of 6–13 repeats while STTR10 had a median size of 11 and a mid-range of 9–12 repeats. We also analyzed the frequency and allele size of the top 10 MLVA types that existed over the 7 years. The top 10 MLVA types vary from year to year from less than 1% to over 20%, which account for 32.6% of total isolates (Table 2). The range of alleles for STTR5, STTR6 and STRTR10 were 7–12, 6–13, and 8 to 13 respectively, all of which were in the mid-range. The predominance of mid-range repeats in the population suggests a direction evolution of the VNTRs toward mid-range repeats.

**Table 2:** **Percentage of predominant MLVA types from 2007 to 2014**.

**MLVA type**	**Year**							
	**2007**	**2008**	**2009**	**2010**	**2011**	**2012**	**2013**	**2014**
2-7-6-12-212	0.41	8.55	17.94	23.46	5.61	0.78	5.70	0.96
2-7-7-11-212	1.21	6.21	17.89	1.03	6.13	8.88	4.69	2.54
2-7-6-11-212	1.10	2.26	8.50	3.70	13.88	6.90	2.78	5.17
2-8-7-8-212	14.17	2.98	0.87	0.10	8.13	3.64	2.17	1.01
2-8-13-10-112	2.02	8.31	2.34	0.62	2.66	1.61	2.98	0.52
2-7-7-12-212	0.0	0.32	4.61	2.36	1.52	2.56	1.21	0.52
2-7-6-13-212	0.0	0.81	1.18	5.04	3.14	0.72	0.76	0.61
2-10-11-8-212	0.0	0.08	0.06	0.10	1.09	0.18	1.62	4.37
2-7-8-11-212	1.21	0.48	0.69	0.92	0.67	2.33	1.31	1.18
2-12-10-10-212	0.0	0.08	0.19	0.31	2.99	0.89	0.05	0.09

### *In vitro* experimental evolution to confirm directional mutations

**Figure 2:**
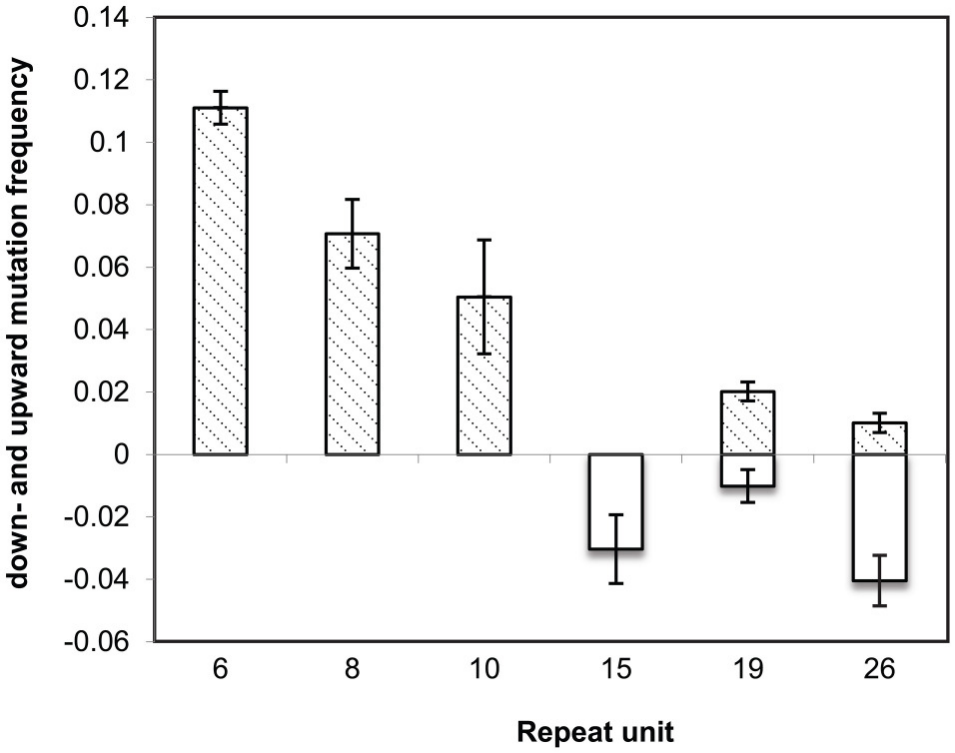
**Allele-specific mutation frequencies observed in MLVA types 2-15-8-10-212 and 2-26-6-19-112 at three VNTR loci: STTR5, STTR6, and STTR10 ***in vitro*** experimental evolution**. In each repeat size, top bars depict upward mutations (insertion) while bottom bars correspond to downward mutations (deletion). Where observed frequencies are larger than zero, standard errors are plotted as error bars.

To confirm the directional mutability of the VNTR loci, an *in vitro* evolution experiment was carried out using two MLVA types 2-15-8-10-212 and 2-26-6-19-112 represented by L2162 and L2191, respectively. The latter carried an extreme length allele of 26 and 19 repeats for STTR5 and STTR10, respectively. After 50 generations of passaging in three replicates, 34 events were observed in 32 colonies (32/198, 16%), of which 33 mutation events were changes due to single repeats (either insertion or deletion) while one mutation involved change of two repeats (Table 3). The repeat changes fitted the general VNTRs mutation model proposed by Vogler et al. (2006), which predicts that the majority of mutations involve single repeats. Four and three mutation events with one repeat unit deletion were observed in allele 26 and allele 15 (both STTR5), respectively (Figure 2). In contrast, alleles 6 (STTR6), 8 (STTR6) and 10 (STTR10) were all mutated by one repeat unit insertion and observed in 11, seven and five of the 99 colonies for each MLVA type sampled, respectively. These observations suggest that long VNTRs are likely to mutate to shorter ones, and short VNTRs are likely to gradually become longer. Allele 19 (in STTR10) did not exhibit the expected deletion pattern with two insertion events and only one deletion event.

**Table 3:** **Mutations observed at MLVA loci in two MLVA types of ***S***. Typhimurium**.

**Repeat unit (Locus)**	**Insertion**	**No. of insertion**	**Deletion**	**No. of deletion**
6 (STTR6)	+1	11	NA	0
8 (STTR6)	+1	7	NA	0
10 (STTR10)	+1	5	NA	0
15 (STTR5)	NA	0	−1	3
19 (STTR10)	+1/+2	1/1	−1	1
26 (STTR5)	−1	1	−1	4

*NA, mutation was not observed*.

### Relationship between VNTR changes and genomic SNPs in single locus variant MLVA types

**Figure 3:**
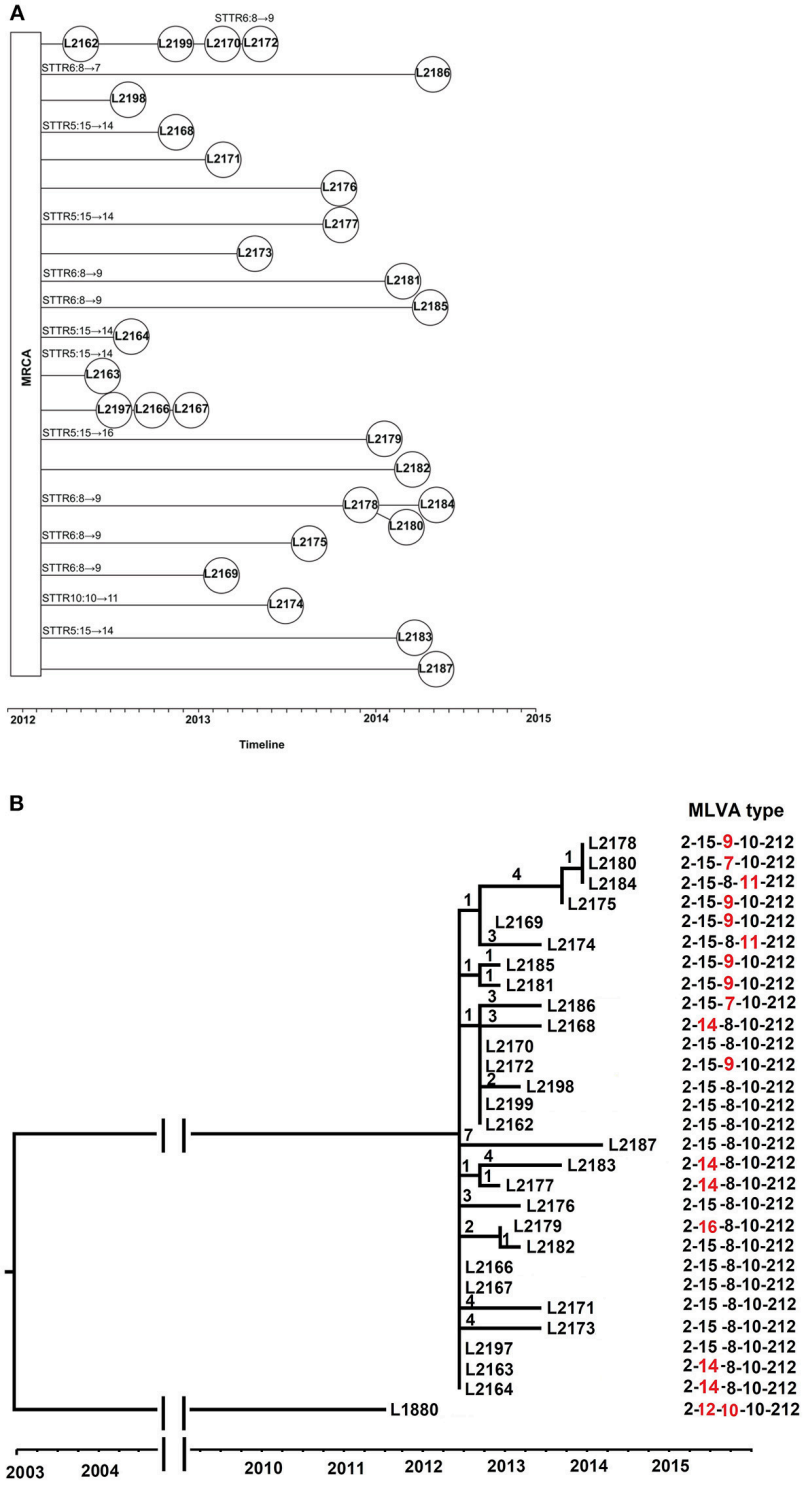
**The phylogenomic relationship and evolutionary time of 28 ***S***. Typhimurium isolates from MLVA type 2-15-8-10-212 and its related MLVA types**. **(A)** The VNTR changes and timeline of 28 *S*. Typhimurium evolved from most recent common ancestor (MRCA). The isolates were connected based on the phylogeny and isolation date. The VNTR changes of each locus for some isolates were labeled upon the lines. **(B)** Phylogeny of 2-15-8-10-212 and its related MLVA types. The phylogenetic tree was visualized and edited by FigTree v1.4.2, which was based on single nucleotide polymorphisms (SNPs) identified by mapping to the reference genome of *S*. Typhimurium LT2. The timeline was indicated below the tree. The MLVA type 2-12-10-10-212 (strain L1880) was used as background isolates, with three and two repeat differences in STTR5 and STTR6 compared with 2-15-8-10-212, respectively. The number on the branches corresponds to the number of SNP difference. Isolates are labeled by their isolation date with strain number. The MLVA type was indicated with the differing locus highlighted in red.

To elucidate the relationship of VNTR change and SNP differences at genome level, we sequenced 28 isolates from 2012 to 2014, from a novel emerging MLVA type, 2-15-8-10-212 and five single locus variant MLVA type at any of the 3 locus, STTR5, STTR6, and STTR10. We chose 2-15-8-10-212 as the main type as the number of this MLVA type (550 isolates) was higher than all of isolates of the other 5 related MLVA types selected and it contained the mid-range size allele for STTR5 (15 repeats) and STTR6 (8 repeats) (Figure S1). We only examined 1 VNTR difference as one allelic change is the most frequent event and more relevant to public health investigations of point source outbreaks (Dimovski et al., 2014; Ahlstrom et al., 2015). We selected 1–6 isolates per MLVA type with matching timeframe as shown in Table S1. All isolates were from eastern Sydney so that the findings are more relevant to local epidemiology as outbreak detection by MLVA is based on spatial and temporal clustering. These isolates were recovered between 20 and 695 days after the initial identification of the first 2-15-8-10-212 isolate (L2162). The VNTR changes and isolation date of the selected 28 *S*. Typhimurium isolates are shown in Figure 3A.

In total, there were 48 SNPs among the 28 isolates (Table S2). Single nucleotide differences were observed as short as 8 days apart (between isolates L2197 and L2198). Identical isolates were found as far as 151 days apart. Compared with the five strains located near the root of the tree (L2163, L2164, L2166, L2167, and L2197), the isolates showed 1–7 single nucleotide differences. The largest number of single nucleotide differences were found in L2187 with 7 SNPs.

Of the 48 SNPs observed, 43 and 5 SNPs were located in coding regions and integenic regions respectively. The majority of the coding region SNPs (30 out of 43) were non-synonymous SNPs (Table 4). Each of the nsSNPs was located in a different gene, most of the gene functions are related to metabolism. The majority of the nsSNPs were only found in a single isolate, suggesting that they were likely to be transient. nsSNPs present singularly in individual isolates have been observed in other *S*. Typhimurium isolates with no apparent adaptive values. For example, Octavia et al. (2015b) analyzed the genomic variation of serial *S*. Typhimurium isolates in human infection related with prolonged carriage and found that the majority of SNPs observed were located in coding regions, most of which were nsSNPs in the genes involved with metabolism. Four nsSNPs were present in 2 or more phylogenetically related isolates including one nsSNP in *adrA* among 4 isolates (L2175, L2178, L2180, and L2184), one nsSNP in STM1543 among 6 isolates (L2169, L2174, L2175, L2178, L2180, and L2184) and one nsSNP in *iroC* between 2 isolates (L2179 and L2182). One nsSNP in *ccmH* was present in all 2-15-8-10-212 isolates except L2171. Three of the four genes are involved in virulence or adaptation. *adrA* encoding diguanylate cyclase plays a role in biofilm formation (García et al., 2004), STM1543 is involved in swarming motility and virulence (Bogomolnaya et al., 2014), while *iroC* is involved in iron acquisition (Crouch et al., 2008).

**Table 4:** **Non-synonymous SNPs in the isolates analyzed**.

**Isolate**	**Gene**	**Locus**	**Product**	**Amino acid change**
L2176		STM0285	Putative inner membrane protein	D → N
L2175/2178/2180/2184	*adrA*	STM0385	Diguanylate cyclase/phosphodiesterase domain 1	P → S
L2185	*hscC*	STM0659	Putative molecular chaperone, DnaJ family	E → K
L2173	*ybjZ*	STM0942	Putative ABC superfamily transport protein	A → V
L2171	*ycaJ*	STM0962	Paral putative polynucleotide enzyme	E → K
L2183	*pepT*	STM1227	Putative transcriptional regulator	Q → Stop
L2169/2174/2175/2178/2180/2184		STM1543	PhoPQ-regulated protein	A → S
L2173	*srfC*	STM1595	SsrAB activated protein	A → V
L2171	*mukB*	STM0994	Chromosome partition protein	K → N
L2187	*erfK*	STM2015	L,D-transpeptidase	V → A
L2176	*pduF*	STM2037	Propanediol diffusion facilitator	Y → D
L2187	*yehU*	STM2159	Two component sensor kinase	G → D
L2183		STM2273	Dehydratase	G → R
L2187	*asrC*	STM2550	Anaerobic sulfite reductase subunit C	I → S
L2173		STM2767	DNA/RNA helicase	E → G
L2179/2182	*iroC*	STM2774	ABC transporter ATP-binding protein	D → G
L2174		STM2922	3-polyprenyl-4-hydroxybenzoate decarboxylase	F → S
L2174		STM3142	Periplasmic ferrichrome-binding protein	L → M
L2176	*arcB*	STM3328	Aerobic respiration control sensor histidine kinase	D → Y
L2186	*gltB*	STM3330	Glutamate synthase subunit alpha	F → L
L2186	*codB*	STM3333	Cytosine permease	L → M
L2186	*nanK*	STM3336	N-acetylmannosamine kinase	D → E
L2176	*argR*	STM3360	Arginine repressor	P → S
L2171	*acrE*	STM3390	Multidrug efflux protein	L → P
L2182	*yiaB*	STM3659	Inner membrane protein	M → I
L2183		STM3784	PTS system mannitol/fructose-specific transporter subunit IIA	S → L
All except for L2171	*ccmH*	STM3812	Heme lyase subunit	T → I
L2187	*hemX*	STM3936	Uroporphyrinogen III C-methyltransferase	M → I
L2185	*pfkA*	STM4062	6-phosphofructokinase	S → R
L2168		STM4552	Inner membrane protein	P → L

A phylogenetic tree was inferred from the SNPs (Figure 3B). Some isolates were indistinguishable by SNP-based typing but were differentiated by MLVA, and vice versa. For instance, L2178, L2184, and L2180 had the same genome type but different MLVA types. Similarly, two isolates L2163 and L2164 with MLVA type 2-14-8-10-212 and three isolates L2197, L2166, and L2167 with MLVA type 2-15-8-10-212 were found to be genomically identical although the two MLVA types differed only by one VNTR.

The SNPs were mapped on to the branches of the phylogenetic tree (Figure S2). There were no reverse or parallel changes for the SNPs present in more than one isolate, indicating that none of the SNPs was derived from recombination. Maximum parsimony analysis was also performed on the dataset and found that the homoplasy index was zero, further confirming no reverse or parallel changes for the SNPs present in the dataset.

### A coalescent model of joint distribution of variation of VNTRs and SNPs

**Figure 4:**
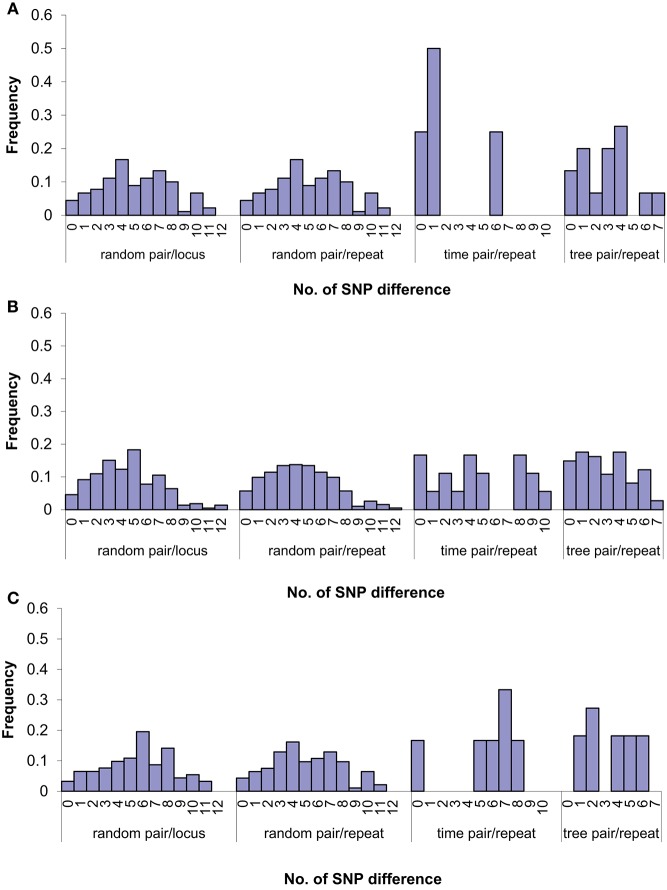
**Relationship between SNP and VNTR difference by pair-wise comparisons of isolates for zero (A)**, one **(B)**, and two **(C)** VNTR repeat/locus changes. Random pair/locus: random pair-wise comparisons between isolates with given VNTR locus difference; random pair/repeat: random pair-wise comparisons between isolates with given VNTR repeat difference; time pair/repeat: pair-wise comparisons between isolates with given VNTR repeat difference based on the same time frame within a 1-month window; tree pair/repeat: pair-wise comparisons between isolates at the root and the ones on each respective branches in the SNP tree with given VNTR repeat difference. The bars are represented by the proportion of number of samples at each number of SNPs difference.

We further examined the relationship of SNPs and VNTRs by pairwise comparisons (Figure 4). The SNP difference per VNTR locus or per VNTR repeat ranged from 0 to 12. The majority of the SNP differences per VNTR repeat fell within 6 SNPs. When we restricted pairwise comparisons to a 1 month window which grouped 28 isolates into 11 monthly intervals (Table S1), the majority of differences were 4 SNPs or fewer. We also examined the SNP differences per repeat along the phylogenetic lineages which have 9 branches (Figure 3B) and compared the isolates at the root with the ones in the respective branches in a pairwise fashion. We found that the range was smaller, ranging from 0 to 7 SNPs. We then compared isolates with 2 VNTR differences. The range of SNP differences was similar to those differing by 0 or 1 (Figure 4), highlighting that there is no relationship between VNTR and SNP differences beyond one VNTR locus difference.

We used the coalescent model to examine the virtual ratio of mutation rates between VNTR changes and genome SNP variation statistically. Using the 28 genomes that contained 48 SNPs and 7 VNTR mutation events (5 mutation events from 2-15-8-10-212 and 2 mutation events from 2-15-9-10-212 in L2184 and L2180), we can obtain a maximum likelihood estimate of per genome per generation mutation rate in effective population size *N* unit for SNPs and VNTRs of 0.96 (95% confidence interval 0–2.00) and 6.57 (95% confidence interval 1.58–11.56), respectively. The relative rate was estimated to be 1 VNTR change to 6.9 (95% confidence interval 0.65–13.1) SNP changes (Figure S3).

### Estimation of SNP mutation rate

The SNP mutation rate estimated by Path-O-gen (Rambaut et al., 2016) gave a substitution rate of 6.0 × 10^−7^ site^−1^ year^−1^ or 3 SNPs per genome per year while BEAST (Drummond et al., 2012) gave a substitution rate of 6.83 × 10^−7^ site^−1^ year^−1^ (3.14–9.62 × 10^−7^, 95% HPD) or 3 SNPs genome per year. This rate is similar to a previous estimate from a *S*. Typhimurium phage type DT135a outbreak in Australia (Hawkey et al., 2013), but faster than that *S*. Typhimurium outbreaks causing invasive typhoid-like disease in Africa (1–2 SNPs genome^−1^ year^−1^; Okoro et al., 2012).

## Discussion

In this study, we examined the behavior of VNTR mutations using a large set of MLVA data from public health laboratory surveillance over an 8 year period and found that mid-range of repeat units of 8–14 in STTR5, 6–13 in STTR6 and 9–12 in STTR10 were always predominant in the population (>50%) and extreme repeat units were rare. We attribute this phenomenon to the directional mutability of the VNTRs. To further verify this observation, we analyzed the published Belgium dataset from 2010 to 2012, which also used the same MLVA typing scheme (Wuyts et al., 2013). We found that most of the MLVA types (129 out of 137) with extreme allele length found in 2010 disappeared in 2011 or 2012, which were likely to have converted to MLVA types with median size. In contrast, the top five prevalent MLVA types all contain alleles in the mid-size range and persisted throughout the whole period, accounting for 24.87% of all isolates (Table S3). This directional mutability was also confirmed in our *in vitro* evolution experiments.

We examined other published *Salmonella* datasets including additional five VNTR loci from *S*. Typhimurium (Hiley et al., 2014) and four VNTR loci from *S*. Paratyphi A (Yao et al., 2014), as well as three VNTR loci from a *Mycobacterium avium* dataset (Ahlstrom et al., 2015) to determine whether directional mutability applies to VNTRs in other *Salmonella* serovars or other bacterial species. These datasets are relatively small and consist of a few hundred isolates. Although all had small number of alleles and one allele dominates, directional mutability is apparent with all dominant alleles falling within mid-range repeats (Figures S4–S6).

The directional mutability toward the median range of VNTR repeats might be associated with the stabilizing mechanism to maintain the biological function. Removal of extreme length alleles benefits the organism as the function of the gene is retained. Analogous to single base mutations, extreme length VNTR mutations may be deleterious. For instance, STTR5 is located between two domains of YohM, a protein involved in nickel and cobalt resistance (Rodrigue et al., 2005). STTR3 is located between a signal peptide and a polypeptide chain in BigA, a surface-exposed virulence protein (Curiao et al., 2016). The extreme length in STTR5 or STTR3 may affect the stability of YohM and weaken the resistance to nickel and cobalt or reduce the virulence of BigA by interfering with the signal peptide and/or affecting the protein secretion. Likewise, for STTR9, STTR6, and STTR10 which are located in intergenic regions, expansion of tandem repeats may affect the binding of the transcription factor in the intergenic region (Shimada et al., 2008). Therefore, directional mutability of a VNTR may be imposed by selection pressure or functional constraints.

However, directional mutations toward the mid-range size may be due to intrinsic mechanisms that are not related to functional selection pressure. In eukaryotes such as yeast, *Drosophila* and humans, mutability of microsatellite has been observed to be dependent on allele size (Falush and Iwasa, 1999; Harr and Schlötterer, 2000; Xu et al., 2000). In *D. melanogaster* long microsatellites (>15 repeats) were extreme rare and have downward mutation bias and can only be maintained for short times (Harr and Schlötterer, 2000), while in humans it was found that insertion mutation rate is relatively constant while deletion mutation rate increases exponentially with increase of allele size (Xu et al., 2000). A latter study of six microsatellites of human Y-Chromosome showed high frequency of upward mutations for low allele size and vice versa (Jochens et al., 2011).

The top 10 MLVA types varied from year to year from less than 1% to over 20%, which account for 32.6% of total isolates and some persisted over several years as endemic MLVA types (Table 2). We found that all endemic MLVA types contained mid-range repeats for STTR5, STTR6, and STRTR10. The directional mutability of VNTRs may partly explain why some MLVA types become prevalent and endemic. Endemic types are partly a result of VNTRs mutating toward mid-size range rather than selective forces driving the increase of certain MLVA types. However, directional mutability also leads to genetic heterogeneity of MLVA types as a result of homoplasy. That is, the same MLVA types may not always be closely related genetically. This homoplasy may impact on the use of MLVA to determine whether cases belonging to the same outbreak in public health investigations. Caution should be exercised in the interpretation of MLVA based local/short term epidemiology. Studies in *M. avium* also showed similar limitations that MLVA cannot be used for national epidemiology with the predominant MLVA type INMV-2 being dispersed throughout the tree in multiple clusters and clearly originated from separated sources (Ahlstrom et al., 2015).

Conversely, isolates that differ by one VNTR could be closely related or identical by genomic SNPs. Our analysis of 28 isolates from 6 MLVA types differing by one VNTR showed that 5 and 3 isolates with the same genome type were differentiated by three and two MLVA types, respectively. Dimovski et al. (2014) also found that two MLVA types with one repeat difference have the same genome type in an *S*. Typhimurium outbreak in Tasmania, Australia. Therefore, these observations have implications for outbreak investigations. In our current practice, identification of a potential outbreak is based on identical MLVA types. Our data suggest the criteria may be too stringent and isolates differ by one VNTR locus, in particular, those differ by a single repeat may be identical genomically by SNPs.

Pairwise SNP differences between 28 *S*. Typhimurium isolates ranged from 0 and 12 per VNTR locus (Figure 4). The majority of the differences per VNTR mutation fell within 6 SNPs. Our modeling of the joint distribution of VNTR variation and SNP variation gave a ratio of 6.9 SNPs per VNTR change. Our results were also consistent with a previous report that showed one VNTR change corresponded to 0–10 chromosomal SNPs for the two rapidly changing VNTR loci, STTR5 and STTR6 (Dimovski et al., 2014).

There was no significant relationship between VNTR variation and SNP variation. Similar findings were also found in *M. avium* strains (Ahlstrom et al., 2015). In that study, genome comparison of 124 isolates from 11 different MLVA types showed that the range of pairwise SNP difference between isolates were similar from 1 to 2 SNPs to 239–240 SNPs, regardless of whether they belonged to the same or different MLVA types (Ahlstrom et al., 2015). Interestingly, the study by Eyre et al. (2013) which compared MLVA and genome sequencing for the tracking of *Clostridium difficile* transmission events in hospitals demonstrated a good concordance of MLVA and SNPs in 58 of 61 clustered cases. They found a genomic difference of ≤ 2 SNPs correlated well in transmission determination with a summed VNTR repeat difference of ≤ 10 (out of 7 loci). However, concordance decreased significantly over larger differences.

Elucidation of the relationship of SNP differences per VNTR locus difference also supports relaxing the criteria for the use of MLVA for outbreak investigations. When we restricted pairwise comparison to 1 month window, the majority of the isolates were separated by 4 or fewer SNPs. Based on the relationship of SNP and VNTR changes and our previous proposal that 4 SNPs may be used as a cut-off for “ruling in” an isolate to belong to the same outbreak (Octavia et al., 2015a), the majority of the isolates with 1 VNTR repeat change should be ruled in as outbreak-related isolates for epidemiological investigation. The current consensus definition relying on identical MLVA types to detect community outbreaks should be relaxed to include 1 VNTR repeat difference in the three rapidly evolving loci STTR5, STTR6, and STTR10 to increase sensitivity. This suggestion is consistent with a previous proposition (Dimovski et al., 2014) which was based on a study of 203 isolates from a series of linked outbreaks of *S*. Typhimurium in Tasmania, Australia.

## Conclusion

The prevalence of endemic types of *S*. Typhimurium is influenced by the directional mutability of VNTRs. It also leads to genetic heterogeneity of MLVA types due to homoplasy. Coalescent modeling of MLVA and SNP variation provided estimates of the mutation rates of VNTRs and SNPs with the ratio of 1 VNTR change to 6.9 SNP changes. However, there is no relationship between SNP and VNTR differences. When only one VNTR repeat difference was considered, the majority of pairwise SNP difference between isolates were 4 SNPs or fewer. Based on this observation and our previous findings of SNP differences of outbreak isolates (Octavia et al., 2015a), inclusion of *S*. Typhimurium isolates with one VNTR repeat difference from the three most variable loci in clusters of potentially related organisms should increase sensitivity of public health laboratory surveillance of *S*. Typhimurium outbreaks.

## Author contributions

Conceived and designed the experiments: SF, SO, and RL. Performed the experiments: SF, SO, QW, and VS. Genomic sequencing was performed by CT. Data analysis and draft of manuscript were performed by SF, SO, MT, and RL. All authors approved the final version of the manuscript for submission.

### Conflict of interest statement

The authors declare that the research was conducted in the absence of any commercial or financial relationships that could be construed as a potential conflict of interest.
