# Supplementary material for: Evolution of Variable Number Tandem Repeats and Its Relationship with Genomic Diversity in Salmonella Typhimurium
Source: Front Microbiol. 2016 Dec 26;7:2002. doi: 10.3389/fmicb.2016.02002 (PMC5183578; doi:10.3389/fmicb.2016.02002)
Supplement: Supplementary file 1 [file DataSheet1.pdf]

Figure S1 The prevalence of 2-15-8-10-212 and its related MLVA types between 2012 and 2014

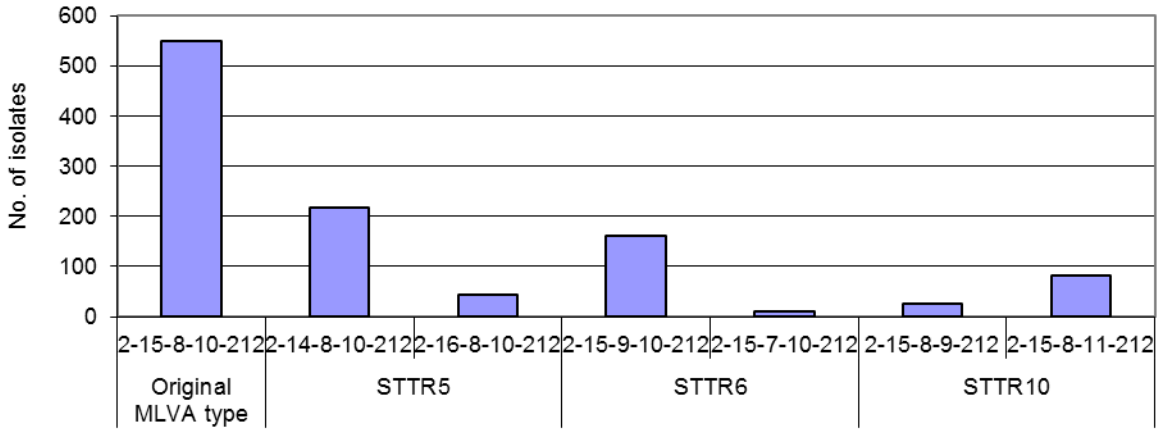

Figure S2 Maximum parsimony tree of *S*. Typhimurium genomes of 2-15-8-10-212 and its related MLVA types, which was based on single nucleotide polymorphisms (SNPs) identified by mapping to the reference genome of *S*. Typhimurium LT2. The MLVA type 2-12-10-10-212 (strain L1880) was used as background isolates. Scale bar indicates the number of SNPs.

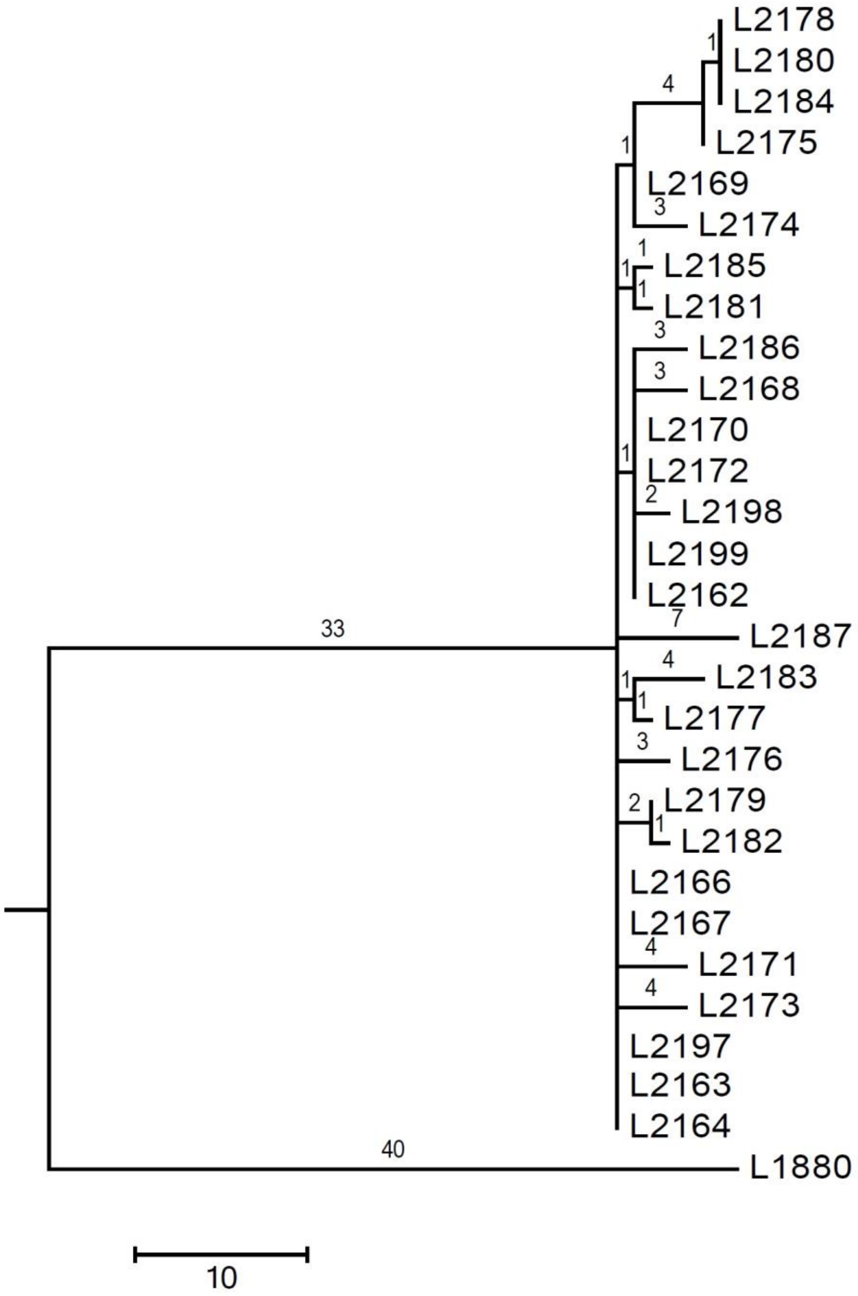

Figure S3 Negative log likelihood surface with contours. The figure was plotted using the following parameter: s = 48, v = 7, n = 27. The surface was computed as − log[P (v, s; λ, r)] which was reparametrised the model with λ and r =λ/μ. The smallest number in the log likelihood surface indicated the values for λ and r in x-axis and y-axis (λ=6.57, r=6.9), respectively.

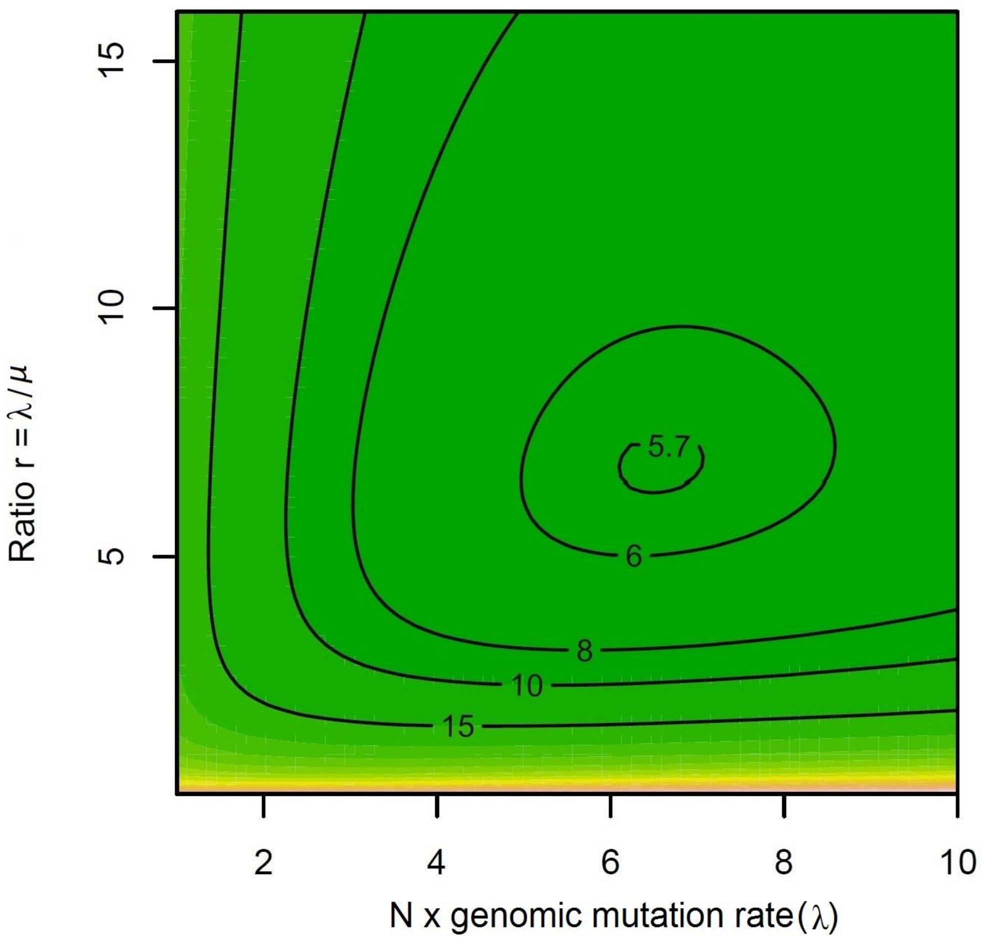

Figure S4 Distribution of allele size in STTR1 (A), STTR7 (B), STTR8(C), STTR11 (D), STTR12 (E), and ST3 (F) among 206 *S*. Typhimurium strains. The data were obtained from Hiley et al. (2014).

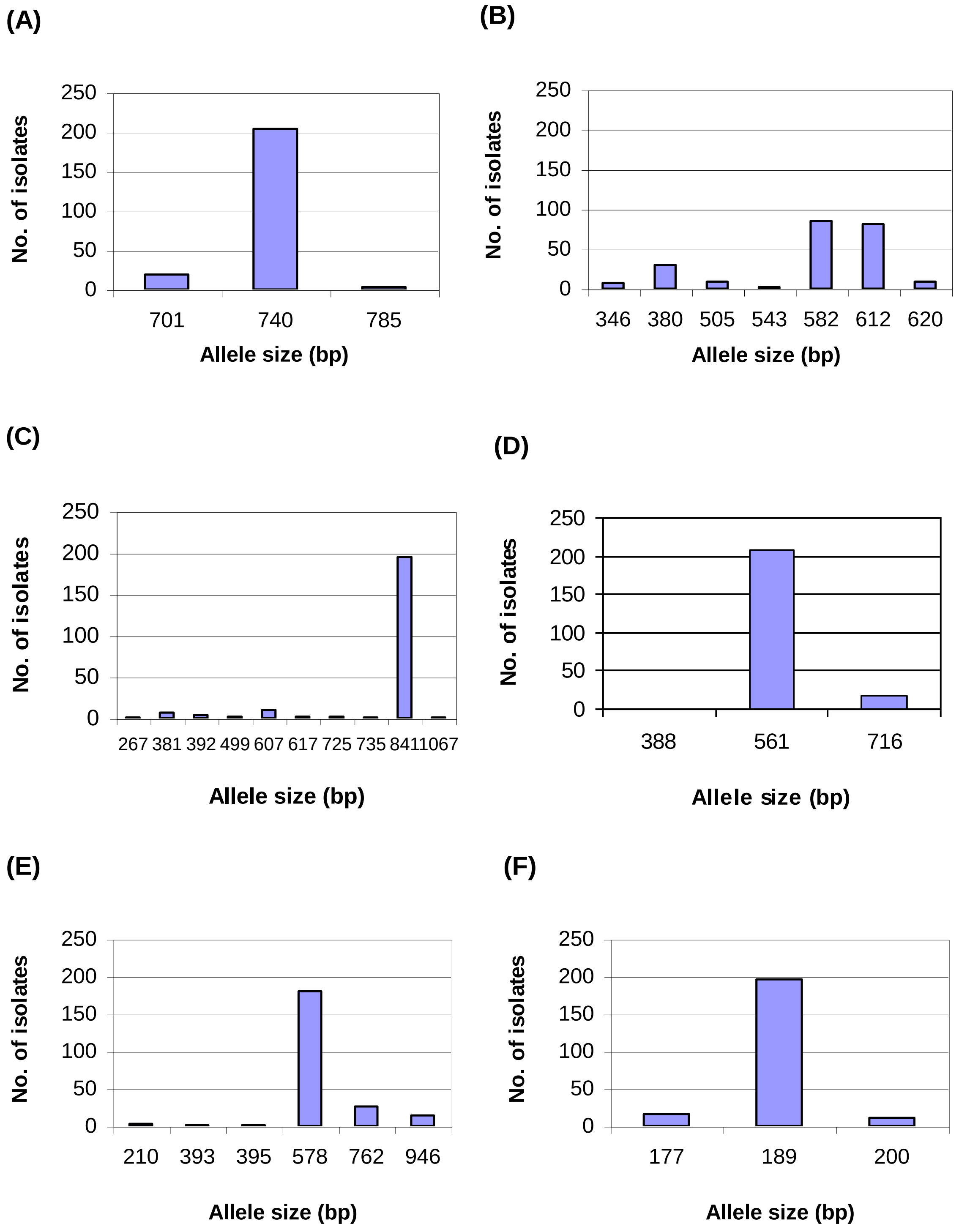

Figure S5 Distribution of repeat unit in TR27 (A), TR41 (B), TR51(C), TR43 (D), among 215 *Salmonella Paratyphi* A strains. Other loci that have less than three alleles were excluded from analysis. The data were obtained from: Yao et al. (2014)

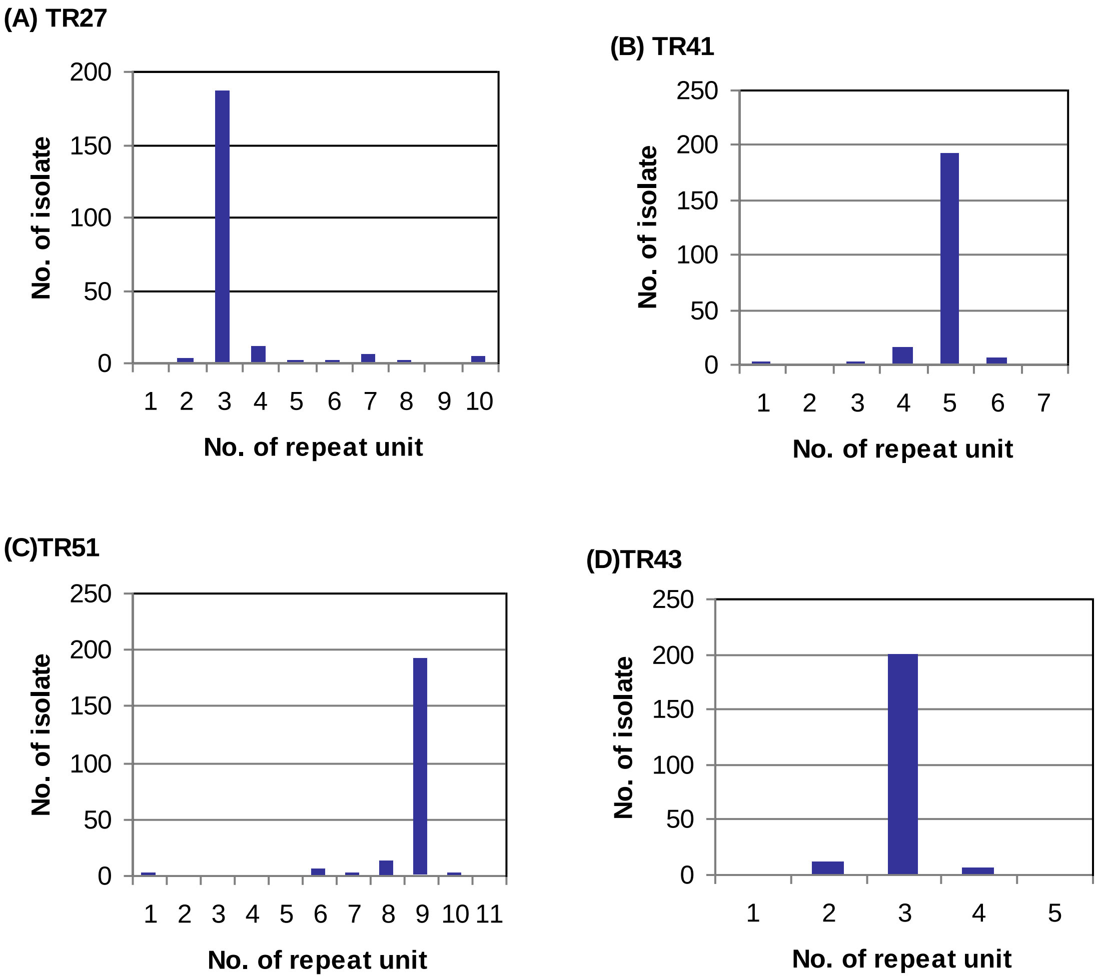

Figure S6 Distribution of repeat unit in locus 292 (A), 25 (B), X3 (C), among 527 *Mycobacterium avium* strains. Other loci that have less than three alleles were excluded from analysis. The data was obtained from Ahlstrom et al. (2015).

(A) Locus 292

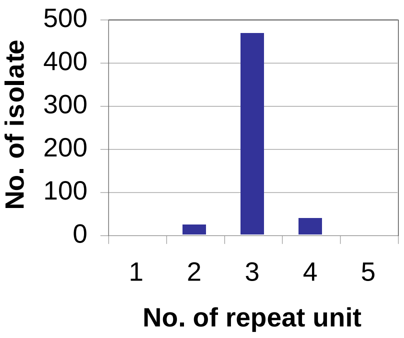

(B) Locus 25

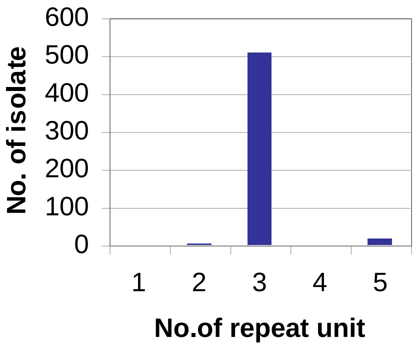

(C) Locus X3

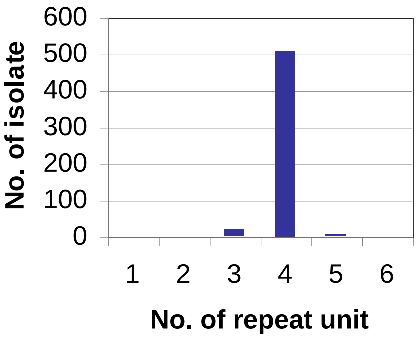

Table S1 Selection of strains based on the match of isolation date

No. of groups	2-15-8-10-212 strain	Isolation date	MLVA with one repeat difference strain	Isolation date
1	L2162	4/06/2012	L2163	24/06/2012
2	L2198	3/08/2012	L2164	3/08/2012
	L2197	26/7/2012		
	L2166	7/09/2012		
3	L2167	11/11/2012	L2168	22/11/2012
	L2199	19/12/2012		
4	L2170	16/02/2013	L2169	4/02/2013
	L2171	25/02/2013		
5	L2173	29/04/2013	L2172	15/04/2013
			L2174	19/05/2013
6	N/A		L2175	30/08/2013
7	L2176	25/11/2013	L2177	25/11/2013
			L2178	22/12/2013
8	N/A		L2179	11/01/2014
9	L2182	23/02/2014	L2180	27/01/2014
			L2181	27/01/2014
			L2183	26/02/2014
10	N/A		L2184	11/03/2014
11	L2187	29/04/2014	L2185	1/04/2014
			L2186	23/04/2014

N/A: not applicable

TableS2 Single nucleotide polymorphisms of 28 sequenced isolates*

Position	2163/2164/2166/2167/2197	2162	2168	2173	2169	2174	2175	2170	2171	2172	2176	2177	2178/2180/2184	2179	2181	2182	2183	2185	2186	2187	2199	2198
325940	G	.	.	.	.	.	.	.	.	.	A	.	.	.	.	.	.	.	.	.	.	.
438201	C	.	.	.	.	.	T	.	.	.	.	.	T	.	.	.	.	.	.	.	.	.
526796	A	.	.	.	.	.	.	.	.	.	.	.	.	.	.	.	.	.	.	T	.	.
530831	C	.	.	.	.	.	T	.	.	.	.	.	T	.	.	.	.	.	.	.	.	.
723663	C	.	.	.	.	.	.	.	.	.	.	.	.	.	.	.	.	T	.	.	.	.
920553	C	T	T	.	.	.	.	T	.	T	.	.	.	.	.	.	.	.	T	.	T	T
956366	T	.	.	.	.	.	.	.	.	.	.	.	.	C	.	C	.	.	.	.	.	.
1021656	C	.	.	T	.	.	.	.	.	.	.	.	.	.	.	.	.	.	.	.	.	.
1042529	G	.	.	.	.	.	.	.	A	.	.	.	.	.	.	.	.	.	.	.	.	.
1122417	G	.	.	.	.	.	.	.	.	.	.	A	.	.	.	.	A	.	.	.	.	.
1279396	G	.	A	.	.	.	.	.	.	.	.	.	.	.	.	.	.	.	.	.	.	.
1314599	C	.	.	.	.	.	.	.	.	.	.	.	.	.	.	.	T	.	.	.	.	.
1612632	G	.	.	.	.	.	.	.	.	.	.	A	.	.	.	.	.	.	.	.	.	.
1619408	C	.	.	.	A	A	A	.	.	.	.	.	A	.	.	.	.	.	.	.	.	.
1684389	C	.	.	T	.	.	.	.	.	.	.	.	.	.	.	.	.	.	.	.	.	.
1082476	G	.	.	.	.	.	.	.	T	.	.	.	.	.	.	.	.	.	.	.	.	.
1902253	G	.	.	.	.	.	.	.	.	.	.	.	.	.	.	.	.	.	.	T	.	.
2026899	G	.	.	A	.	.	.	.	.	.	.	.	.	.	.	.	.	.	.	.	.	.
2096561	A	.	.	.	.	.	.	.	.	.	.	.	.	.	.	.	.	.	.	G	.	.
2116015	A	.	.	.	.	.	.	.	.	.	C	.	.	.	.	.	.	.	.	.	.	.
2255358	C	.	.	.	.	.	.	.	.	.	.	.	.	.	.	.	.	.	.	T	.	.
2300420	G	.	.	.	.	.	A	.	.	.	.	.	A	.	.	.	.	.	.	.	.	.
2306245	G	.	.	.	.	.	.	.	.	.	.	.	T	.	.	.	.	.	.	.	.	.

2377428	C	.	.	.	.	.	.	.	.	.	.	.	.	.	.	.	G	.	.	.	.	.
2406119	C	.	.	.	.	.	.	.	.	.	.	.	.	.	.	.	.	.	.	T	.	.
2489009	G	.	.	.	.	.	.	.	.	.	.	.	.	.	.	.	.	.	.	.	.	T
2689118	T	.	.	.	.	.	.	.	.	.	.	.	.	.	.	.	.	.	.	G	.	.
2716939	G	.	.	.	.	.	.	.	.	.	.	.	.	.	.	.	T	.	.	.	.	.
2786424	A	.	.	.	.	.	G	.	.	.	.	.	G	.	.	.	.	.	.	.	.	.
2908472	T	.	.	C	.	.	.	.	.	.	.	.	.	.	.	.	.	.	.	.	.	.
2919431	A	.	.	.	.	.	.	.	.	.	.	.	.	G	.	G	.	.	.	.	.	.
3064070	T	.	.	.	.	C	.	.	.	.	.	.	.	.	.	.	.	.	.	.	.	.
3307096	G	.	.	.	.	T	.	.	.	.	.	.	.	.	.	.	.	.	.	.	.	.
3491775	C	.	.	.	.	.	.	.	.	.	A	.	.	.	.	.	.	.	.	.	.	
3495294	T	.	.	.	.	.	.	.	.	.	.	.	.	.	.	.	.	.	C	.	.	.
3502627	C	.	.	.	.	.	.	.	.	.	.	.	.	.	.	.	.	.	A	.	.	.
3505301	G	.	.	.	.	.	.	.	.	.	.	.	.	.	.	.	.	.	C	.	.	.
3528235	C	.	.	.	.	.	.	.	.	.	T	.	.	.	.	.	.	.	.	.	.	.
3561435	T	.	.	.	.	.	.	.	C	.	.	.	.	.	.	.	.	.	.	.	.	.
3844691	C	.	.	.	.	.	.	.	.	.	.	.	.	.	.	T	.	.	.	.	.	.
3983985	G	.	.	.	.	.	.	.	.	.	.	.	.	.	.	.	A	.	.	.	.	.
4012634	A	.	.	.	.	.	.	.	G	.	.	.	.	.	.	.	.	.	.	.	.	.
4026380	C	.	A	.	.	.	.	.	.	.	.	.	.	.	.	.	.	.	.	.	.	.
4143775	C	.	.	.	.	.	.	.	.	.	.	.	.	.	.	.	.	.	.	T	.	.
4273259	C	.	.	.	.	.	.	.	.	.	.	.	.	.	A	.	.	A	.	.	.	.
4348727	C	.	.	.	.	T	.	.	.	.	.	.	.	.	.	.	.	.	.	.	.	.
4810404	C	.	T	.	.	.	.	.	.	.	.	.	.	.	.	.	.	.	.	.	.	.
4818306	C	.	.	.	.	.	.	.	.	.	.	.	.	.	.	.	.	.	.	.	.	T

*** Dot indicates the identical nucleotide with 2163/2164/2166/2167/2197**

Table S3 The dynamics of the number of MLVA types in 2010, 2011 and 2012 in Belgium

MLVA Profile	2010	2011	2012	Total
3-13-10-NA-211	41	25	18	84
3-12-10-NA-211	38	25	18	81
3-12-9-NA-211	28	17	28	73
3-12-11-NA-211	27	17	28	72
3-13-9-NA-211	12	11	19	42
3-13-8-NA-211	7	7	21	35
3-14-11-NA-311	11	15	7	33
3-15-11-NA-311	10	15	5	30
3-15-10-NA-311	6	12	10	28
3-15-12-NA-311	4	8	14	26
3-16-10-NA-311	14	4	7	25
3-12-8-NA-211	2	11	10	23
3-14-10-NA-311	15	4	0	19
3-14-10-NA-211	10	4	2	16
3-14-9-NA-211	5	4	7	16
3-8-9-NA-211	3	5	6	14
3-12-12-NA-211	2	4	7	13
3-12-10-NA-509	1	4	7	12
3-14-8-NA-211	3	1	7	11
3-17-11-NA-211	3	1	7	11
3-14-11-NA-211	1	7	2	10
3-17-11-NA-311	1	0	7	8
3-27-12-NA-311	1	0	7	8
3-5-11-NA-211	1	7	0	8
3-11-11-NA-211	2	3	1	6
3-9-7-8-211	5	1	0	6
4-9-10-10-111	1	5	0	6
3-14-12-NA-311	1	0	4	5
3-9-10-NA-211	1	3	1	5
2-12-7-9-212	3	0	1	4
3-10-8-9-211	1	3	0	4
3-13-13-16-311	4	0	0	4
3-13-7-NA-211	1	1	2	4
3-14-18-14-311	3	1	0	4
3-15-10-23-410	4	0	0	4
3-15-13-4-311	3	1	0	4
3-10-11-7-211	1	1	1	3
3-10-14-7-211	3	0	0	3
3-10-7-9-211	3	0	0	3
3-11-8-NA-211	3	0	0	3

3-12-15-17-311	1	1	1	3
3-12-7-NA-211	3	0	0	3
3-12-9-NA-NA	1	0	2	3
3-13-11-NA-211	3	0	0	3
3-13-8-NA-311	2	1	0	3
3-14-19-16-NA	3	0	0	3
3-15-10-23-311	3	0	0	3
3-15-12-14-311	1	0	2	3
3-15-8-NA-311	3	0	0	3
3-15-9-NA-311	1	1	1	3
3-16-9-NA-311	1	2	0	3
4-13-17-13-211	1	2	0	3
4-9-10-13-211	3	0	0	3
4-9-10-9-211	3	0	0	3
4-9-12-9-211	3	0	0	3
2-9-21-12-212	2	0	0	2
2-9-NA-12-212	1	1	0	2
3-10-7-NA-311	2	0	0	2
3-11-8-NA-310	2	0	0	2
3-11-NA-NA-211	2	0	0	2
3-12-18-26-NA	2	0	0	2
3-12-5-12-311	2	0	0	2
3-12-9-NA-310	2	0	0	2
3-13-10-NA-311	2	0	0	2
3-13-11-NA-311	2	0	0	2
3-13-12-NA-311	1	0	1	2
3-13-8-NA-NA	1	0	1	2
3-14-10-15-311	1	1	0	2
3-14-10-NA-210	2	0	0	2
3-14-14-22-311	1	0	1	2
3-14-16-14-311	2	0	0	2
3-14-16-23-311	1	1	0	2
3-14-19-12-311	1	1	0	2
3-14-19-16-311	2	0	0	2
3-14-8-NA-105	1	1	0	2
3-14-9-NA-311	2	0	0	2
3-15-10-NA-410	2	0	0	2
3-15-11-NA-211	2	0	0	2
3-15-11-NA-410	2	0	0	2
3-15-13-14-311	1	1	0	2
3-15-13-14-410	2	0	0	2
3-15-16-22-311	2	0	0	2

3-15-8-NA-211	1	1	0	2
3-16-16-28-311	2	0	0	2
3-16-2-NA-311	1	0	1	2
3-17-10-NA-311	2	0	0	2
3-18-10-NA-311	2	0	0	2
3-18-9-NA-211	1	1	0	2
4-10-16-10-211	2	0	0	2
4-14-10-7-211	1	1	0	2
6-8-15-NA-211	1	1	0	2
6-9-13-NA-211	2	0	0	2
3-13-19-15-311	1	0	0	1
1-10-11-7-NA	1	0	0	1
1-13-10-NA-112	1	0	0	1
1-13-NA-NA-14	1	0	0	1
1-14-15-22-311	1	0	0	1
1-15-10-23-410	1	0	0	1
2-10-20-8-312	1	0	0	1
2-11-11-10-411	1	0	0	1
2-11-11-11-312	1	0	0	1
2-12-17-11-212	1	0	0	1
2-12-6-9-212	1	0	0	1
2-12-7-8-212	1	0	0	1
2-13-18-7-212	1	0	0	1
2-14-9-9-212	1	0	0	1
2-15-NA-NA-105	1	0	0	1
2-16-12-9-105	1	0	0	1
2-20-12-12-212	1	0	0	1
2-6-4-8-212	1	0	0	1
2-7-10-9-210	1	0	0	1
2-7-11-9-309	1	0	0	1
3-10-12-NA-211	1	0	0	1
3-10-13-NA-211	1	0	0	1
3-10-15-7-211	1	0	0	1
3-10-7-10-211	1	0	0	1
3-10-NA-7-NA	1	0	0	1
3-11-10-NA-211	1	0	0	1
3-11-15-25-311	1	0	0	1
3-11-16-25-311	1	0	0	1
3-11-27-18-NA	1	0	0	1
3-11-3-NA-211	1	0	0	1
3-11-5-NA-111	1	0	0	1
3-11-6-8-211	1	0	0	1

3-11-8-8-211	1	0	0	1
3-11-9-NA-211	1	0	0	1
3-12-10-13-211	1	0	0	1
3-12-10-NA-310	1	0	0	1
3-12-15-NA-211	1	0	0	1
3-12-9-NA-311	1	0	0	1
3-13-10-NA-111	1	0	0	1
3-13-10-NA-310	1	0	0	1
3-13-13-15-311	1	0	0	1
3-13-13-16-NA	1	0	0	1
3-13-14-14-311	1	0	0	1
3-13-14-15-311	1	0	0	1
3-13-14-24-410	1	0	0	1
3-13-16-23-410	1	0	0	1
3-13-5-12-311	1	0	0	1
3-13-5-13-311	1	0	0	1
3-13-6-23-311	1	0	0	1
3-13-6-NA-211	1	0	0	1
3-13-NA-11-311	1	0	0	1
3-14-0-14-311	1	0	0	1
3-14-0-22-311	1	0	0	1
3-14-10-14-105	1	0	0	1
3-14-10-15-NA	1	0	0	1
3-14-10-24-211	1	0	0	1
3-14-12-25-211	1	0	0	1
3-14-12-NA-410	1	0	0	1
3-14-13-14-410	1	0	0	1
3-14-14-NA-311	1	0	0	1
3-14-15-14-311	1	0	0	1
3-14-16-22-410	1	0	0	1
3-14-17-24-311	1	0	0	1
3-14-18-15-311	1	0	0	1
3-14-19-13-311	1	0	0	1
3-14-20-12-212	1	0	0	1
3-14-22-14-311	1	0	0	1
3-14-6-NA-211	1	0	0	1
3-14-8-NA-310	1	0	0	1
3-14-9-NA-410	1	0	0	1
3-15-1-19-311	1	0	0	1
3-15-10-24-410	1	0	0	1
3-15-10-NA-105	1	0	0	1
3-15-11-NA-105	1	0	0	1

3-15-12-NA-211	1	0	0	1
3-15-12-NA-NA	1	0	0	1
3-15-13-15-311	1	0	0	1
3-15-13-NA-510	1	0	0	1
3-15-14-24-311	1	0	0	1
3-15-8-12-311	1	0	0	1
3-15-9-NA-410	1	0	0	1
3-15-NA-NA-111	1	0	0	1
3-16-10-NA-410	1	0	0	1
3-16-11-NA-311	1	0	0	1
3-16-11-NA-410	1	0	0	1
3-16-12-14-311	1	0	0	1
3-17-10-NA-410	1	0	0	1
3-17-9-NA-211	1	0	0	1
3-19-5-NA-311	1	0	0	1
3-23-NA-NA-311	1	0	0	1
3-8-15-21-NA	1	0	0	1
3-9-6-8-11	1	0	0	1
3-9-8-9-211	1	0	0	1
4-10-12-10-211	1	0	0	1
4-11-11-7-211	1	0	0	1
4-13-9-NA-211	1	0	0	1
4-14-15-8-211	1	0	0	1
4-15-6-7-211	1	0	0	1
4-16-14-8-211	1	0	0	1
4-17-17-7-211	1	0	0	1
4-22-12-7-211	1	0	0	1
4-8-12-8-211	1	0	0	1
4-8-12-9-211	1	0	0	1
4-8-13-8-211	1	0	0	1
5-10-11-9-211	1	0	0	1
5-9-19-9-211	1	0	0	1
5-9-6-9-211	1	0	0	1
6-8-15-NA-211	1	1	0	2
6-9-13-NA-211	2	0	0	2
